# Evidence for poplar PtaPLATZ18 in the regulation of plant growth and vascular tissues development

**DOI:** 10.3389/fpls.2023.1302536

**Published:** 2023-12-21

**Authors:** Claire Guérin, Marc Behr, Julie Sait, Adeline Mol, Mondher El Jaziri, Marie Baucher

**Affiliations:** Laboratoire de Biotechnologie Végétale, Université libre de Bruxelles, Gosselies, Belgium

**Keywords:** PLATZ, transcription factor, poplar, vascular tissue development, lignin, secondary growth

## Abstract

**Introduction:**

Plant A/T-rich protein and zinc-binding protein (PLATZ) are plant-specific transcription factors playing a role in plant development and stress response. To assess the role of PLATZs in vascular system development and wood formation in poplar, a functional study for *PtaPLATZ18*, whose expression was associated with the xylem, was carried out.

**Methods:**

Poplar dominant repressor lines for *PtaPLATZ18* were produced by overexpressing a *PtaPLATZ18-SRDX* fusion. The phenotype of three independent transgenic lines was evaluated at morphological, biochemical, and molecular levels and compared to the wild type.

**Results:**

The PtaPLATZ18-SRDX lines showed increased plant height resulting from higher internode length. Besides, a higher secondary xylem thickness was also evidenced in these dominant repression lines as compared to the wild type suggesting an activation of cambial activity. A higher amount of lignin was evidenced within wood tissue as compared to the wild type, indicating an alteration in cell wall composition within xylem cell types. This latter phenotype was linked to an increased expression of genes involved in lignin biosynthesis and polymerization.

**Discussion:**

The phenotype observed in the PtaPLATZ18-SRDX lines argues that this transcription factor targets key regulators of plant growth and vascular tissues development.

## 1 Introduction

PLATZ are plant-specific transcription factors (TFs) that bind A/T-rich DNA sequences. They are characterised by two conserved domains required for their zinc-dependent DNA-binding, C-X_2_-H-X_11_-C-X_2_-C-X_(4-5)_-C-X_2_-C-X_(3-7)_-H-X_2_-H and C-X_2_-C-X_(10-11)_-C-X_3_-C (Nagano et al., 2001). Some PLATZ proteins have been localized both in the nucleus, which is consistent with their function as transcriptional regulators, and in the cytoplasm, suggesting a role in cellular signaling pathways (So et al., 2015; Li et al., 2017; Kim et al., 2018; Zhou and Xue, 2020; Li et al., 2021; Han et al., 2022; Wai et al., 2022; Zhao et al., 2022). PLATZ TFs are central elements in protein accumulation by acting at two distinct regulation levels. On the one hand, they regulate the biosynthesis of transfer RNAs (tRNAs) and 5S ribosomal RNA (5S rRNA) through protein-protein interactions within the RNA polymerase III complex (Pol III) (Li et al., 2017; Wang et al., 2018). On the other hand, several PLATZ can control transcription by binding A/T-rich sequences in a Pol II-dependent process (Kim et al., 2018). PLATZ have been identified either as transcriptional activators (Kim et al., 2018; Hu et al., 2023) or repressors (Nagano et al., 2001; Liu et al., 2020; Zhao et al., 2022; Zhang et al., 2023). The size of the PLATZ family varies between species. For instance, 12 *PLATZ* genes have been identified in Arabidopsis (Wang et al., 2018), 17 in *Zea mays* (Wang et al., 2018), and 18 in *Populus trichocarpa* (Ma et al., 2023).

The characterization of several *PLATZ* from monocot and dicot plants has highlighted their role in the regulation of a large panel of stress responses and/or developmental processes. First, the expression of several *PLATZ* is induced under abiotic stress, and they play a role in regulating abiotic stress tolerance. For instance, in Arabidopsis, AtPLATZ1 and AtPLATZ2 have been identified as regulators of seed desiccation tolerance (González-Morales et al., 2016). AtPLATZ1/AIN1 is also involved in ABA-mediated inhibition of root elongation by modulating ROS homeostasis (Dong et al., 2021). AtPLATZ2 is involved in the regulation of stress tolerance as lines overexpressing *AtPLATZ2* had an increased sensitivity to salt stress (Liu et al., 2020). AtPLATZ4 was found to promote drought tolerance by directly repressing *Plasma membrane Intrinsic Protein 2;8* (*PIP2;8*) as well as by regulating genes involved in ABA signalling (Liu et al., 2023). In poplar, *PtPLATZ1-4* and *PtPLATZ8-9* are involved in cadmium tolerance as evidenced by heterologous expression in yeast and overexpression in poplar (Ma et al., 2023).

Second, PLATZ were found to regulate developmental processes including seed filling, floral development, and organ as well as plant growth. For instance, the *Z. mays* semi-dominant mutant *floury 3* (*fl3*) showed a defect in endosperm development (Li et al., 2017). FL3 (also named ZmPLATZ12) interacts with proteins of the Pol III complex including RPC53, TbBRF1, NRPC2, and TFC1, and the *fl3* mutant has defects in Pol III activity as shown by reduced accumulation of 5S rRNA and several tRNAs (Li et al., 2017; Zhao et al., 2020). Another maize PLATZ, ZmPLATZ2, was found to directly bind to the *ZmSSI* starch synthetic gene promoter and when *ZmPLATZ2* was transiently expressed in maize endosperm or overexpressed in rice, an upregulation of starch synthesis genes was observed (Li et al., 2021). In *O. sativa*, the *Osfl3* CRISPR/Cas9 knockout lines were also impacted in grain development as shown by their reduced grain weight and size (Guo et al., 2022). *FL3* was positively regulated by the *Triticum aestivum* Positive regulator of Grain Size1 (PGS1) basic helix-loop-helix TF through binding to the E-box motif in the *FL3* promoter of both wheat and rice (Guo et al., 2022). The rice GLABROUS GENE 6 (GL6)/SHORT GRAIN 6 (SG6) was found to participate in the Pol III transcription machinery by interacting with the RPC53 and TFC1 subunits to promote cell division in the spikelet hull, which positively controls grain length (Wang et al., 2019; Zhou & Xue, 2020). Finally, the ectopic expression of the soybean *GmPLATZ* in Arabidopsis triggers larger seeds with a 12.5-49% increase in the 1000-seed weight (Hu et al., 2023). On the opposite, these authors showed that the knockout *gmpla/b* soybean mutants had a ~10% lower 100-seed weight and smaller seeds with reduced cell numbers but no difference in seed nutrient composition compared to the control.

The *Vitis vinifera* VviPLATZ1 is involved in the control of female flower morphology as in the CRISPR/Cas9 mutants, female flowers with reflex stamens were observed instead of hermaphrodite flowers in the wild type (WT) (Iocco-Corena et al., 2021). The Arabidopsis *AtPLATZ10* expression was reported to be controlled by the Agamous-mediated biotimer suggesting a role in flower development (Pelayo et al., 2023).

Phenotypic and molecular analyses of Arabidopsis activation-tagged lines and knockout mutants demonstrated that AtPLATZ3 (ORESARA 15, ORE15) controls cell proliferation during growth as well as leaf senescence and directly regulates the genes encoding the TFs GROWTH-REGULATING FACTOR (GRF) 1/4 in a Pol II-dependent process (Kim et al., 2018). Consistently, the expression of *ORE15* peaks in young leaves and progressively decreases during leaf aging (Kim et al., 2018; Jun et al., 2019). In addition, ORE15 was reported to be a regulator of root growth and development as it positively controls root apical meristem size in a process involving auxin and cytokinin signalling-related pathways (Timilsina et al., 2022). Transgenic Arabidopsis overexpressing *AtPLATZ4* exhibited a lower fresh weight than the WT, and it was suggested that AtPLATZ4 acts as a negative regulator of growth by suppressing the expression of *EXP6* and *EXP16 expansin* genes thereby inhibiting the expansion of the cell wall (Liu et al., 2023).

A role of PLATZs in developmental processes linked to perennial growth, such as wood formation has not been reported yet. However, there are indications of the involvement of PLATZ in secondary cell wall (SCW) formation in several plant species. Actually, in hemp and nettle, two species harboring extraxylary fibers with a gelatinous-type SCW, several *PLATZs* were upregulated in thickening bast fibers, either as compared to elongating bast fibers or to the xylem core of the stem (Guerriero et al., 2017; Xu et al., 2019). These genes were among the most highly expressed TF-encoding genes in the investigated tissues, supporting a role in SCW thickening. In addition, a transcriptomic analysis made in *Populus deltoides × P. euramericana* along a gradient from the shoot apex to the fifth internode, where secondary growth is well developed, highlighted the preferential expression of several *PLATZs* in internodes with cells undergoing SCW thickening (Chao et al., 2019). As this expression pattern is comparable to those of well-described regulators of SCW biosynthesis, such as homologs of Arabidopsis *NST2*, *MYB46*, and *MYB83*, these authors suggested a role for some *PLATZ* genes in the transition from primary to secondary growth. Finally, Potri.013G078500 (PLATZ14) was detected in a shotgun analysis of the nuclear-fractionated proteome of stem-differentiating xylem tissue of *P. trichocarpa* (Loziuk et al., 2017).

To assess the role of *PLATZs* in vascular system development and wood formation in poplar (*P. tremula x P. alba*), a functional study for *PtaPLATZ18*, whose expression was associated with the xylem, was investigated. The *PtaPLATZ18* dominant repression (SRDX) lines produced by expressing full-length *PtaPLATZ18* in frame with the *SUPERMAN repression domain X* (*SRDX*) displayed an enhanced plant elongation. Besides, a higher secondary xylem thickness was also evidenced in the dominant repression lines of *PtaPLATZ18* as compared to WT suggesting an activation of cambial activity in the transgenic lines. Finally, a significantly higher amount of lignin was evidenced within wood tissue as compared to the WT, indicating an alteration in cell wall composition within xylem cell types. This report evidences a role for *PtaPLATZ18* in vascular tissues development in poplar, a woody perennial species.

## 2 Materials and methods

### 2.1 *In silico* expression analysis

The RNA-seq data used in this study were retrieved from Sundell et al. (2017). Hierarchical clustering and heatmapping of *P. tremula PtPLATZ* genes were conducted using Mev software (Howe et al., 2011) with Euclidean distance and complete linkage clustering.

### 2.2 Plant material

Both WT and transgenic poplars (*P. tremula* x *P. alba* clone INRA 717-1B4) were grown aseptically and then transferred in soil in the phytotron for three months, as described previously (25°C, 16h day/8h night, 55% humidity) (Baldacci-Cresp et al., 2015).

### 2.3 Cloning of *PtaPLATZ18*, gene constructs, plant transformation, and selection of homogeneous transgenic poplar lines

*PtaPLATZ18* was amplified from *P. tremula* x *P. alba* 717-1B4 cDNA (primers are given in **Supplementary Table 1**), cloned into the pCR BLUNT II TOPO (Life Technologies, Carlsbad, CA, USA) and sequenced. For the *PtaPLATZ18-SRDX* fusion construct, *PtaPLATZ18* was further amplified by PCR using a forward primer containing *att*B1 and a reverse primer containing the sequence encoding both the SRDX (LDLDLELRLGFA) peptide (Hiratsu et al., 2003) and the *att*B2 sequence (**Supplementary Table 1**), and cloned in the pK7WG2D (Karimi et al., 2002) plasmid by Gateway technology (**Supplementary Figure 1**). The binary vector was transformed into poplar *via Agrobacterium tumefaciens* (C58^Rif^PMP90) (Leple et al., 1992). A minimum of 20 independent transgenic lines were produced and the expression level of the *PLATZ18-SRDX* construct was analyzed for 15 of them (**Supplementary Figure 2**). Subsequently, six lines were multiplied *in vitro* and grown for three months in soil in a phytotron (25°C, 16h day/8h night, 55% of humidity), with five biological replicates. Among them, three lines (SRDX2, SRDX19, and SRDX20), showing a significant increase in height, were selected for further characterization (**Supplementary Figure 2**).

### 2.4 Expression profile analyses by RT-qPCR

For the expression profile analysis of *PLATZ* genes in different organs and tissues, three biological replicates and two trees per biological replicate were sampled. Samples from the apex (first 2 cm of the stem), young stem (internodes from 2-7 cm under the apex), young leaves (numbers 10-12 under the first expanded leaf), young roots and lignified roots, as well as secondary vascular tissues from a portion of the stem taken at 15-45 cm below the apex *i.e.* xylem (debarked stem, scratched with a scalpel) and phloem (bark) of 3-month-old WT poplars, were collected and immediately frozen in liquid nitrogen.

For the analysis of SRDX2, SRDX19, and SRDX20 lines, young stem (internode number 1 to 4 under the first expanded leaf) and xylem (debarked stem from 30 to 60 cm from the ground, scratched with a scalpel) were sampled and immediately frozen in liquid nitrogen. Five biological replicates, made of one plant per replicate, were sampled for each SRDX line and the associated WT.

Total RNA was extracted according to Muoki et al. (2012), followed by a DNAse treatment using the TURBO DNA-Free kit (Fisher Scientific), according to the manufacturer’s instructions. For each RNA sample, reverse transcription was performed on 2 μg of total RNA, using the Protoscript II First Strand cDNA Synthesis Kit (New England Biolabs). RT-qPCR were carried out in three technical replicates in a 20 μl volume containing 25 ng of cDNA, 0.5 µl primers (10 µM), and 10 µl of Luna Universal qPCR Master Mix (NEB #M3003) using a thermocycler LightCycler 480 system (Roche). The thermal cycle was set up as follows: pre-incubation at 95°C/1 min, 40 amplification cycles of 95°C/15 s then 60°C/15 s, a melting curve step at 95°C/5 s, 65°C/1 min then heat up to 97°C at 0.11°C/s, and a cooling step to 40°C. Relative gene expression was determined by the 2^-ΔΔ^ *^C^*_T_ method (Livak and Schmittgen, 2001), using adjusted primer efficiencies, and *CDC2* as a reference gene (Baldacci-Cresp et al., 2016) for expression normalization. Monolignol biosynthesis genes were identified by Wang et al. (2018) and the genes putatively involved in monolignol polymerization were identified by Sundell et al. (2017). Genes coding for TFs associated with secondary cell wall biosynthesis in *P. trichocarpa* were taken from Li et al. (2012) (*SND1-A2* and *VND6-C1*) and Payyavula et al. (2022) (*MYB3* and *MYB28*), and primers for corresponding genes in *P. tremula x P. alba* were designed using PrimerQuest. Primer sequences are given in **Supplementary Table 1**.

### 2.5 Plant phenotyping

Poplar plant height was measured each week for three months. Then, mature leaves (numbers 19 to 21 under the first expanded leaf) from WT and transgenic lines were scanned and analyzed to estimate the mean leaf area, using Digimizer (https://www.digimizer.com/). The internode number was counted starting from the first expanded leaf and the internode mean length was determined by dividing plant height by internode numbers. The stem diameter was measured using a calliper at the basis of the stem. A 2 cm sample was retrieved from the internode number 6 and kept at 4°C in 70% ethanol. Stem cross sections (110 µm thick) were made using an HM 650 V vibratome and photographed by a stereomicroscope (Leica M165-FC). The pictures were analyzed using ImageJ (Schneider et al., 2012) to estimate the percentage of bark, xylem, and pith in the stem.

Stem cross sections were subjected to phloroglucinol staining as described in Speer (1987) or to Maüle staining (5 min in 4% KMnO_4_, three water baths, 80 sec in 3% HCl, three water baths, observation under microscope in a drop of Tris-HCl 1M) adapted from Yamashita et al. (2016), then examined and photographed under a wide-field microscope (Axio Observer Z1).

### 2.6 Cell wall residue preparation

The basal part (30 cm long) of the debarked stem was dried at 50°C for 48 h and then ground using a ball mill. One gram of wood powder was mixed with 10 ml of methanol:water (80:20, v/v), sonicated for 10 min, and homogenised for 4 h at room temperature. After 2 min of centrifugation at 4000 g, the pellet was washed two times with ethanol 80% and three times with pure acetone, then dried at 35°C for 24 h. The cell wall residue (CWR) quantification was performed on 300 mg of wood powder, with two technical replicates and three biological replicates per line, and the same protocol as described above. The CWR powder was used as material for both the lignin and cellulose analyses.

### 2.7 Lignin content

The lignin content was determined using the cysteine-assisted sulfuric acid (CASA) method (Lu et al., 2021). Briefly, 10 mg of CWR was fully dissolved with 1 mL of 10% L-cysteine (w/v) in 72% sulfuric acid after an incubation of 1 h at room temperature under agitation. The solution was diluted to 100 mL with pure water and CASA lignin content was evaluated based on the absorbance at 283 nm and an absorption coefficient of 11.23 g^−1^·L·cm^−1^. Five biological replicates and two technical replicates were performed.

### 2.8 Cellulose content

To determine the cellulose content, an Updegraff treatment was performed on the CWR powder. The Updegraff reagent is composed of acetic acid, nitric acid, and pure water in 8:1:2 proportions. 1.5 ml of this reagent was added to the samples and heated to 100°C for 30 min. After a centrifugation of 15 min and 4000 g, the pellet was washed three times with pure water and two times with pure acetone, then dried at 35°C for 24 h.

The cellulose content was determined on 10 mg of the obtained powder, according to the protocol described by Dampanaboina et al. (2021). The equation used here is: $Cellulose\;content(\%CWR)=\frac{[Glu]\cdot A\cdot B}{1.1\cdot m}\cdot 100$ with [Glu] as the glucose concentration (µg/ml), A the dilution factor in anthrone solution (here: 0.5), B the sample volume taken to measure the glucose concentration (µl) (here: 0.1), and m the sample mass (mg).

## 3 Results

### 3.1 Several *PLATZ* are expressed within vascular tissues in poplar

**Figure 1:**
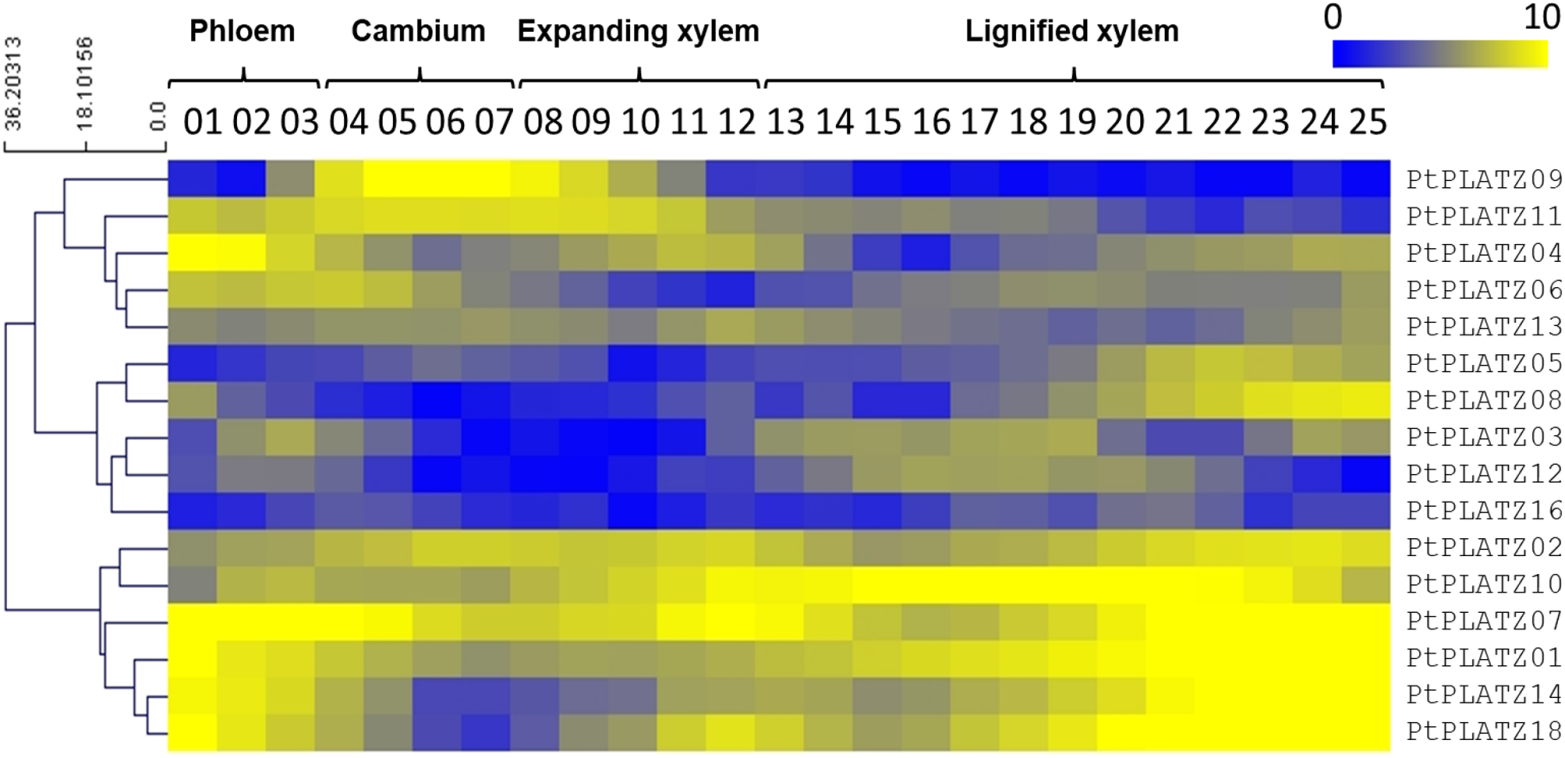
*PLATZ* expression profile as determined by RNA-seq on a series of tangential laser-microdissected sections from phloem to lignified xylem of a 45-year-old *Populus tremula* tree (data from Sundell et al. (2017)). There were no RNA-seq data for *PtPLATZ15* and *PtPLATZ17*.

As a preliminary investigation, the expression pattern of the 18 poplar *PLATZ* genes was studied by retrieving and examining data issued from an RNA-seq analysis performed on a series of laser-microdissected tangential stem sections from phloem to lignified xylem in a 45-year-old *P. tremula* (Sundell et al., 2017). As shown in **Figure 1**, 16 *PLATZs* are expressed within vascular tissues. Some of them are preferentially expressed in the cambial zone (such as *PtPLATZ9*) whereas the expression of others is linked to phloem, xylem, or not differential within the stem. Particularly, six of them (*PtPLATZ1, PtPLATZ2, PtPLATZ7, PtPLATZ10, PtPLATZ14*, and *PtPLATZ18*) display a high expression level in lignified xylem (secondary xylem) suggesting a role for these genes in xylem development and/or SCW formation.

**Figure 2:**
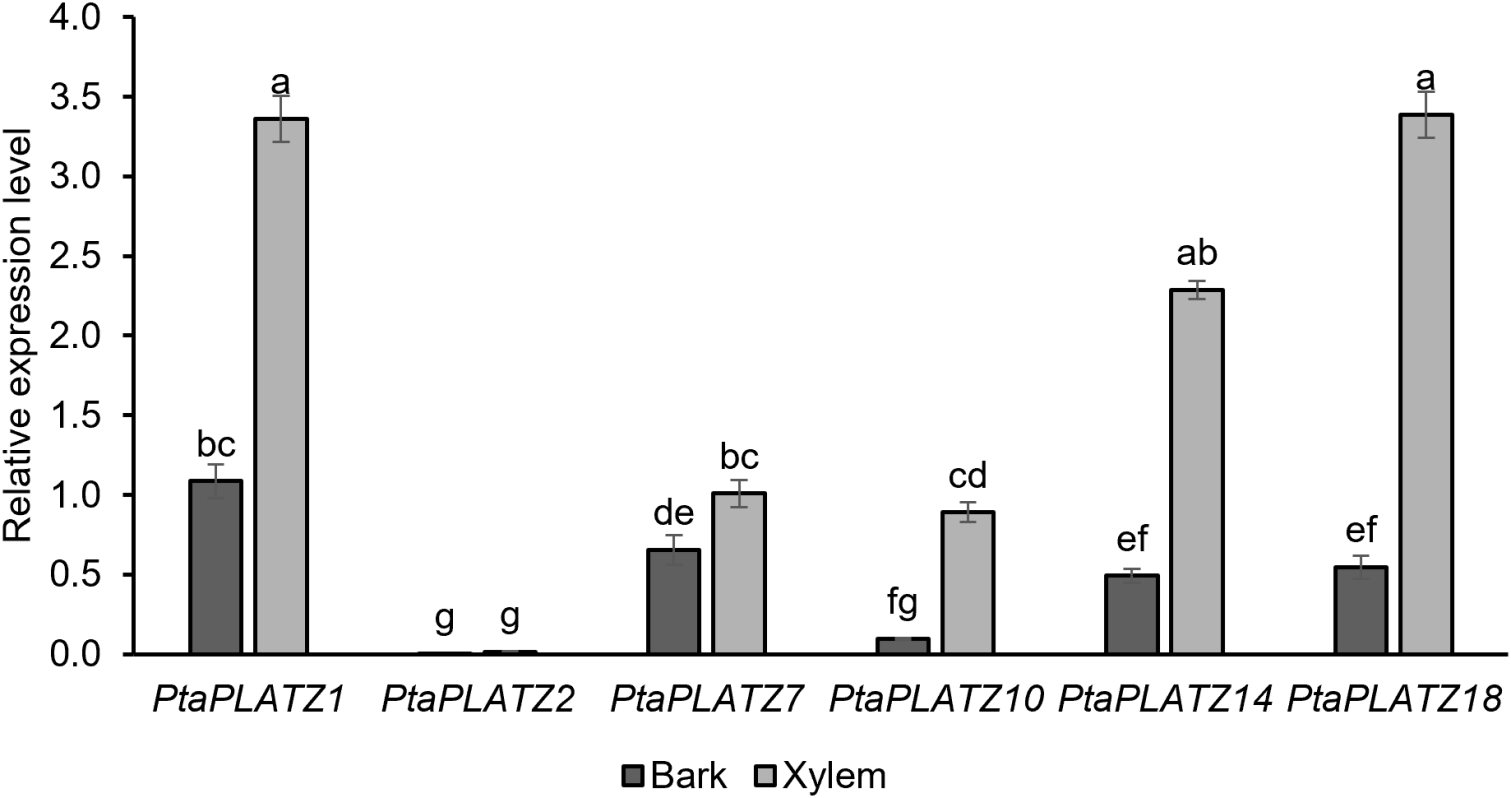
Expression level of six *PtaPLATZ* genes in bark and xylem of the stem of 3-month-old *P. tremula x P. alba* grown in the greenhouse, as measured by RT-qPCR. Bars represent the standard error, from five biological replicates and three technical replicates. Letters indicate the significantly different groups identified by a Kruskal-Wallis test with a Bonferroni correction (*P* < 0.05).

To assess the expression profile of these six genes in the model poplar *P. tremula x P. alba* grown in the phytotron for three months, a RT-qPCR was performed on secondary vascular tissues, *i.e.*, debarked stem (made mainly of secondary xylem) and bark (including phloem). As shown in **Figure 2**, the six *PtaPLATZ* genes showed different expression profiles. Whereas *PtaPLATZ2* was lowly expressed in both tissues, the five other genes, and particularly *PtaPLATZ1, PtaPLATZ14*, and *PtaPLATZ18*, had a higher expression in the xylem compared to bark. To evaluate the specificity of the expression profile of these genes, a complementary expression analysis was made on different organs *i.e.* apex, leaf, young stem, as well as young roots (containing no secondary vascular tissues) and old roots (containing secondary vascular tissues) (**Supplementary Figure 3**). *PtaPLATZ1* was strongly expressed in leaves and roots compared to the apex and young stem, *PtaPLATZ2* had a low but preferential expression in the apex and young roots, *PtaPLATZ7* was expressed at a similar level in all samples, and *PtaPLATZ10* was preferentially expressed in the young root. *PtaPLATZ14* and *PtaPLATZ18* were expressed in all parts but with a 2-3-fold lower relative expression level (**Supplementary Figure 3**) than in the xylem (**Figure 2**). As a case study, *PtaPLATZ18* was selected as a relevant candidate gene for a functional study in the context of vascular tissue development and lignification in poplar.

### 3.2 The dominant repression of *PtaPLATZ18* enhances plant elongation and xylem development

**Figure 3:**
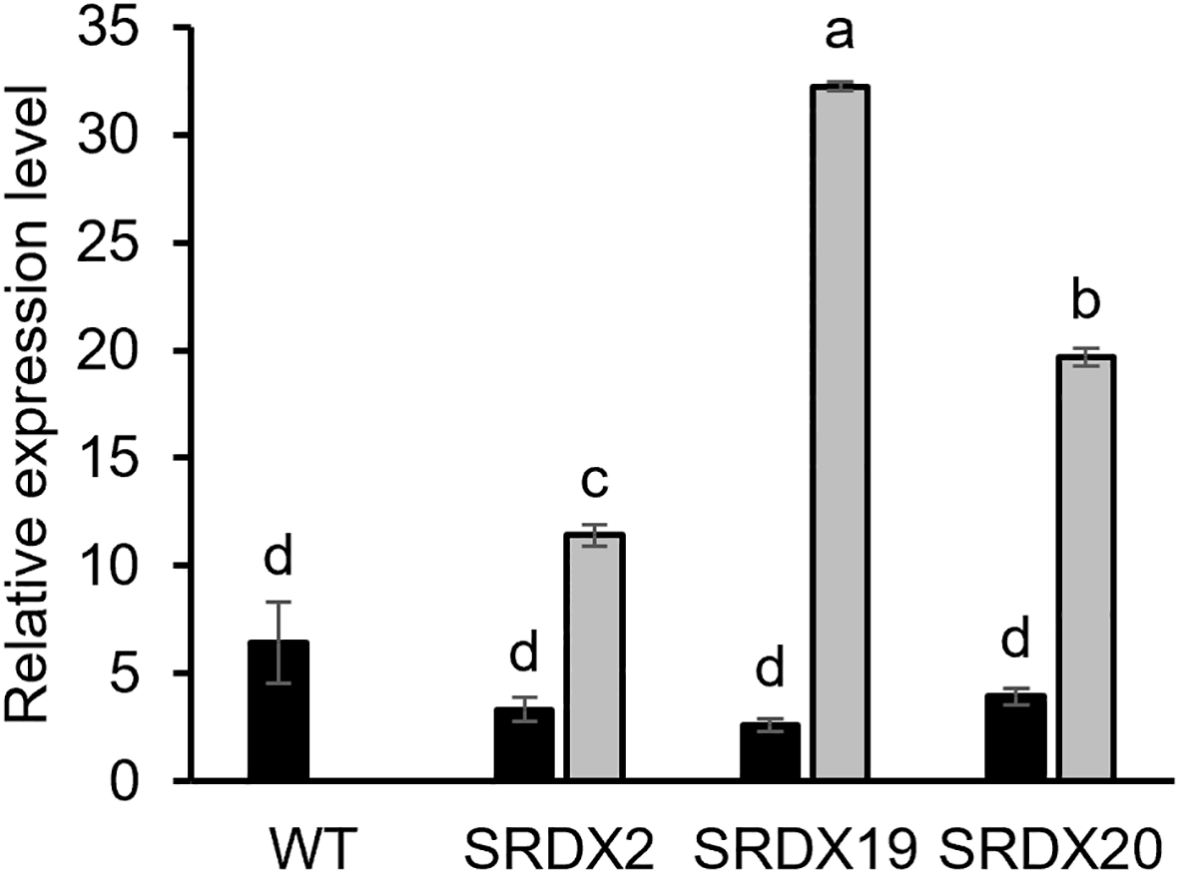
Relative expression level of endogenous *PtaPLATZ18* (black) and *PtaPLATZ18-SRDX* (grey) in the xylem of 3-month-old SRDX and WT lines, as measured by RT-qPCR. Values are the mean and the standard error of five plants (biological replicates) and three technical replicates. The values have been normalized by using the *CDC2* reference gene. Letters indicate the significantly different groups identified by an ANOVA and Tukey, *P* < 0.05.

To explore the function of *PtaPLATZ18* in vascular tissue development and/or lignification, we produced dominant repressor lines for *PtaPLATZ18* by generating poplar lines overexpressing a *PtaPLATZ18-SRDX* fusion. Six transgenic lines expressing *PtaPLATZ18-SRDX* were grown *in vitro* in phytotron for three months. Five of these lines were characterized by a significantly increased height (**Supplementary Figure 2**) and three of them (SRDX2, SRDX19, and SRDX20) were selected for further characterization. The expression level of both endogenous *PtaPLATZ18* and *PtaPLATZ18-SRDX* was analysed in the secondary xylem by RT-qPCR. As shown in **Figure 3**, the endogenous *PtaPLATZ18* expression level was similar for all lines whereas *PtaPLATZ18-SRDX* was highly expressed in the three SRDX lines and not detectable in the WT.

**Figure 4:**
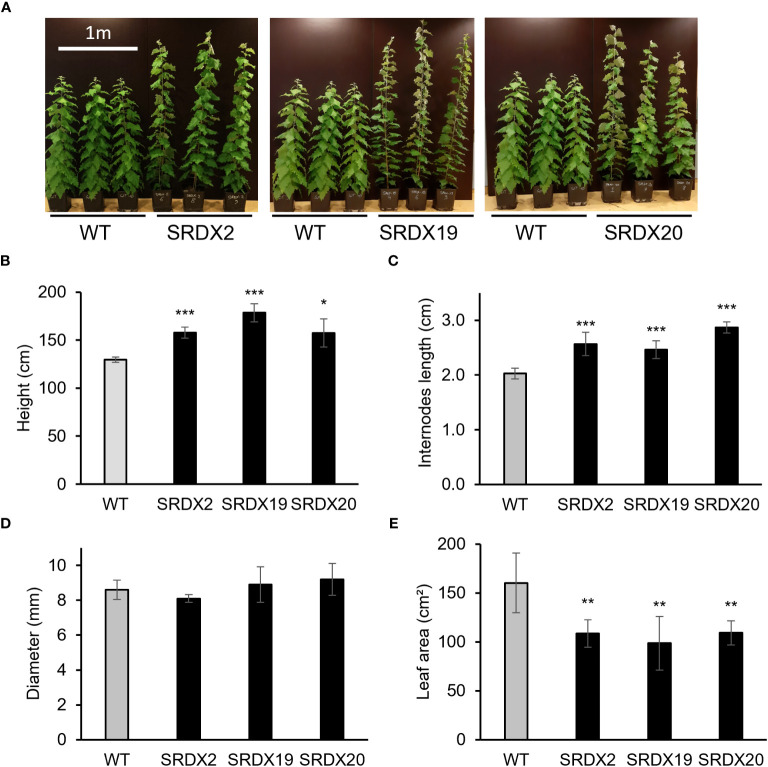
Phenotype of 3-month-old PtaPLATZ18-SRDX poplar lines and the WT. **(A)** Global phenotype. **(B)** Height. **(C)** Internodes mean length. **(D)** Diameter of the basal stem. **(E)** Mean area of leaves numbers 19 to 21. Five biological replicates. Bars = standard deviation. Student *t*-test was performed for each mutant line against the WT. *, *P *< 0.05; **, *P *< 0.01; ***, *P *< 0.001.

**Figure 4** shows the 3-month-old PtaPLATZ18-SRDX lines (SRDX2, SRDX19, and SRDX20) which displayed an enhanced growth compared to the WT (**Figure 4A**) as their height was increased by 22 to 38% (**Figure 4B**). This increase in height was due to an increased mean length of the internodes by 25 to 45% (**Figure 4C**). There was no significant difference in the diameter measured at the basis of the stem of the three transgenic lines and the WT (**Figure 4D**). In addition, SRDX lines exhibit smaller leaves than the WT with a leaf area decreased by 32-38% (**Figure 4E**).

**Figure 5:**
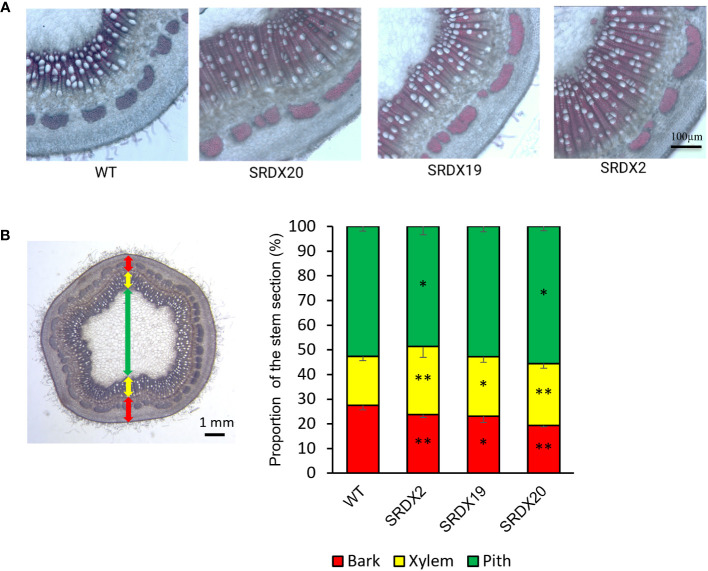
Enhanced xylem formation in 3-month-old PtaPLATZ18-SRDX lines compared to the WT. **(A)** Phloroglucinol-HCl stained stem cross sections (110 µm thickness) of the 6^th^ internode from the apex visualized using bright field microscopy. **(B)** Percentage of bark, xylem, and pith stem sections. Two measures were performed on three stem sections per plant and five plants were studied per line. Student *t*-test was done for each mutant line against the WT. *, *P *< 0.05; **, *P *< 0.01. Bars = standard deviation.

To characterize the SRDX lines at the anatomical level, stem cross sections were realized in the 6^th^ internode below the first expanded leaf and stained with phloroglucinol-HCl to visualize lignified tissues. As shown in **Figure 5A** and **Supplementary Figure 4**, transgenic lines displayed a more developed secondary xylem than the WT. To quantify this observation, bark, xylem, and pith proportions were measured, providing their percentage in the stem sections (**Figure 5B**). A significant increase in the proportion of xylem by 38%, 21%, and 26% was observed in SRDX2, SRDX19, and SRDX20 lines respectively, compared to the WT. This increase in xylem size takes place at the expense of bark, whose proportion decreases by 13%, 16%, and 29% in the three SRDX lines, respectively, compared to the WT.

### 3.3 PtaPLATZ18-SRDX lines have a higher lignin content in the stem xylem than the WT

Subsequently, the impact of the *PtaPLATZ18-SRDX* expression on the cell wall components accumulation was evaluated on CWR produced from wood, taken from the 30 cm basal portion of the stem. As shown in **Table 1**, the amount of recovered CWR was similar for the three SRDX lines and the WT. When compared to the WT, no significant modification was found regarding the cellulose content of the SRDX lines (**Table 1**). On the contrary, they contain a significantly higher lignin content, by 11 to 22%, depending on the lines. A Maüle staining was performed on stem cross section taken at a similar level to those for the phloroglucinol staining to evaluate possible change in lignin composition. This experiment did not reveal a clear difference in qualitative lignin composition between the WT and the SRDX lines (**Supplementary Figure 5**).

**Table 1:** Lignin, cellulose, and CWR content quantified in the WT and the three PtaPLATZ18-SRDX lines.

Line	WT	SRDX2	SRDX19	SRDX20
**Lignin content (% CWR)**	23.05 (± 1.78)	25.75 (± 0.95) *	28.95 (± 2.22) **	27.74 (± 1.41) ***
**Cellulose content (% CWR)**	37.28 (± 6.60)	45.49 (± 8.83)	38.12 (± 5.70)	39.31 (± 9.10)
**CWR (% of wood powder)**	85.14 (± 0.66)	85.42 (± 0.42)	86.16 (± 1.46)	85.14 (± 0.58)

At least three biological replicates and two technical replicates have been performed for each quantification. Data are mean ± standard deviation. *t*-test between the WT and the considered mutant line. *, *P* < 0.05; **, *P* < 0.01; ***, *P* < 0.001.

### 3.4 Higher expression of genes involved in the biosynthesis and polymerization of lignin in the xylem of PtaPLATZ18-SRDX lines

**Figure 6:**
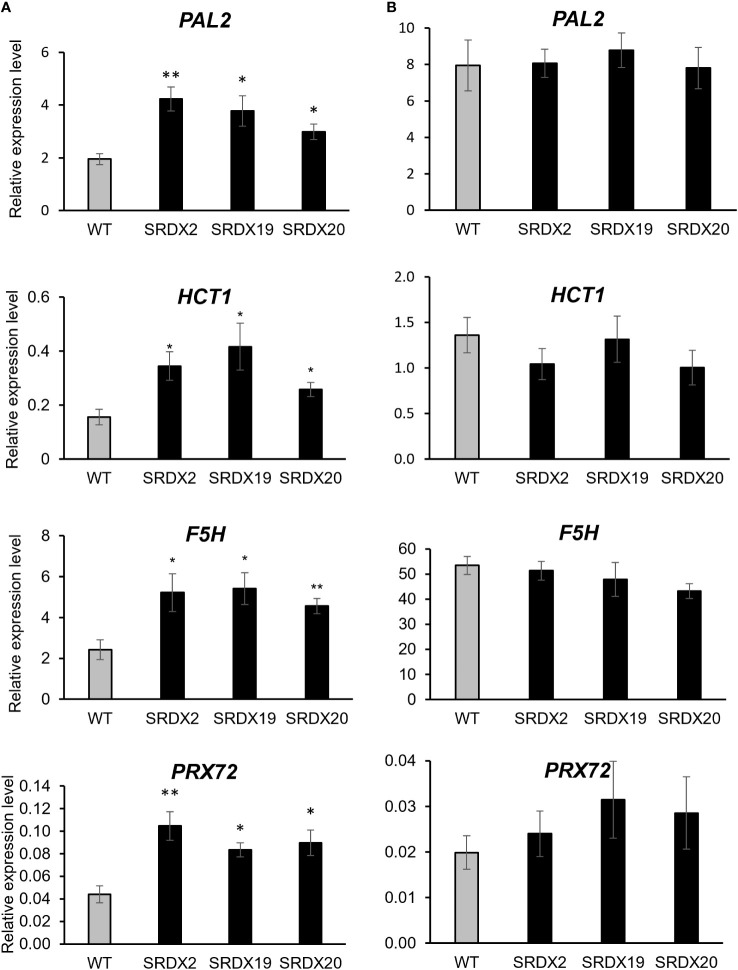
Relative expression level of genes involved in lignin biosynthesis and polymerization in 3-month-old WT and PtaPLATZ18-SRDX lines, as measured by RT-qPCR. **(A)** Young stem. **(B)** Xylem. Value for each line is an average of five plants (biological replicates) and three technical replicates, normalized by the *CDC2* reference gene. Bars = standard error. Student *t*-test was performed for each mutant line against the WT. *, *P* < 0.05; **, *P* < 0.01.

To evaluate whether the increased proportion of secondary xylem tissue and lignin accumulation in the SRDX lines were associated with a raised expression of genes involved in monolignol biosynthesis and/or polymerization, the expression level of several related genes (**Supplementary Table 1**) was subsequently monitored in both whole young stem (at a level comparable to stem sections of **Figure 6**, **Supplementary Figures 4, 5**) and in xylem (at a level comparable the material used for lignin quantification).

In young stems of the three SRDX lines, among the 14 studied genes (**Figure 6A**, **Supplementary Figure 6**), four of them showed a significative ~2-fold increase in their expression level, compared to the WT. These genes include *phenylalanine ammonia-lyase* 2 (*PAL2*), *hydroxycinnamoyl-CoA shikimate transferase 1* (*HCT1*), *ferulate-5 hydroxylase 1* (*F5H1*), and the gene homologous to the *A. thaliana peroxidase 72* (*PRX72*). The expression level of these four genes was further evaluated in xylem. As shown in **Figure 6B**, their expression level was higher than in the young stem and there was no difference between SRDX and WT lines. As there was no differential expression in lignin-related genes in the xylem, one can hypothesize that the higher lignin content in SRDX lines is due to the enhanced expression of lignin biosynthetic and polymerization genes within the young stem, containing mainly developing xylem.

To evaluate which regulation pathway may be altered by the expression of the *PtaPLATZ18-SRDX* construct, the expression level of several genes coding for TFs involved in the regulation of secondary cell wall differentiation was investigated in young stem tissues. Whereas no significant change was monitored for the expression of *SND1-A2*, *MYB3*, or *MYB28*, that of *VND6-C1* was 1.6 to 2.6-fold higher than in the WT, depending on the line (**Supplementary Figure 7**).

## 4 Discussion

Here, we analysed the expression profile of several *PLATZ* genes in different tissues and organs of poplar, and selected *PtaPLATZ18* as a candidate gene regulating secondary growth and/or secondary cell wall formation as it is preferentially expressed within xylem compared to other analysed plant tissues and organs (**Figures 1**, **2** and **Supplementary Figure 3**). We show that the dominant repression of *PtaPLATZ18* triggers phenotypical alterations at morphological, anatomical, and biochemical levels as discussed below.

At the morphological level, dominant repression of *PtaPLATZ18* results in an enhanced growth (by 22-38% depending on the line) associated with increased internode elongation (by 25-45% depending on the line) (**Figure 4**), suggesting that *PtaPLATZ18* controls plant growth and development as already reported for other *PLATZ* genes. For instance, the dominant Arabidopsis *ore15-1D* (*AtPLATZ3*) activation mutant had a 28% increase in plant height when compared to the WT (Kim et al., 2018). Likewise, the overexpression of *T. aestivum PLATZ1-A1* leads to an increased plant height and inversely its mutation caused a reduction of plant height compared to the WT. This gene was found to be the causative gene underlying the gibberellic acid (GA)-sensitive *REDUCED HEIGHT* (*RHT*) *25* dwarfing locus (Zhang et al., 2023). By using yeast two-hybrid and co-immunoprecipitation, these authors showed that PLATZ1-A1 interacts with RHT1, as well as with DELLA, a critical component of the GA growth-stimulating pathway. Recently, *gmpla/b* soybean mutants were described to have reduced leaf size and plant height, which was caused by a reduced internode elongation compared to the WT (Hu et al., 2023). GmPLATZ was found to regulate 3 A-type cyclin (*GmCYCA2;2, GmCYCA2;4a* and *GmCYCA2;4b*) and 3 D-type cyclin (*GmCYCD1;1, GmCYCD4;1* and *GmCYCD6;1*) genes. Besides, this study reported that the expression of *GmGA20OX* was downregulated in the *gmpla/b* mutants and that GmPLATZ activates directly the promoter of *GmGA20OX* through the core element 5’-AATGGGCATT-3’ (Hu et al., 2023).

Stem elongation and growth are known to be regulated by GA level (Davies, 2010). For instance, in poplar, the overexpression of Arabidopsis *GA20 oxidase*, a key enzyme for producing bioactive GA, was reported to increase plant growth and induce xylogenesis (Eriksson et al., 2000; Cho et al., 2019). Actually, 3-month-old transgenic poplars overexpressing the *Pinus densiflora PdGA20ox1* showed an increase of up to ~55% in height with no changes in stem diameter, when compared to the control (Cho et al., 2019).

Further, the dominant repression of *PtaPLATZ18* results in a leaf area decreased by 32-38%, depending on the line (**Figure 4E**). A similar observation was reported for the *ore15-2* knockout mutant (*AtPLATZ3*) characterized by a decreased leaf size mainly caused by an alteration of cell proliferation rather than cell expansion (Kim et al., 2018). ORE15 was found to interact directly with the promoters of *GRF1* and *GRF4*, which are major regulators of plant growth and development including leaf development (Gonzalez et al., 2012).

Pleiotropic effects of elevated GA concentration were noticed in transgenic poplars overexpressing *AtGA20ox1* such as smaller leaf area (Eriksson et al., 2000). In addition, a reduction of 38-40% in leaf area was measured in 2-month-old p35S-PdGA20ox1 poplar lines (Cho et al., 2019).

At the anatomical level, dominant repression of *PtaPLATZ18* results in a significant increase in the proportion of xylem tissue (**Figure 5**) indicating that PtaPLATZ18 plays a role in the formation of secondary growth by controlling cambial activity. Several other genes have been found to promote cambial activity and xylem development in poplar, together with plant height. For instance, the overexpression of *PagRabE1b* encoding a small guanosine triphosphate (GTP)-binding protein leads to an increased xylem width by 27-50% and thicker xylem fiber cell walls (Liu et al., 2021). In another study, the overexpression of *PagSAG101a* induced increased plant height, as well as increased internode number and stem diameter, xylem width (by 5.1-14.7%), and secondary cell wall thickness while opposite phenotypes were observed for *PagSAG10a* knock-out plants (He et al., 2022). As shown by these authors, *PagSAG101a* gene expression is regulated by the PagC3H17 TF. The overexpression of *PagC3H17* (Chai et al., 2014) gave similar results than that of *PagSAG101a*, indicating that the PagC3H17-PagSAG101a module plays a role in the positive gene regulatory network of secondary vascular system development (He et al., 2022). Likewise, overexpression of *UNFERTILIZED EMBRYO SAC12 (UNE12)* encoding a basic helix-loop-helix (bHLH) TF promoted xylem development and increased the thickness of the cell walls and the lignin content (as analyzed by FTIR and Raman microspectrometry) of secondary xylem in poplar together with a reduced plant height, shorter internodes and curled leaves, when compared to the WT (Song et al., 2023).

At the biochemical level, dominant repression of *PtaPLATZ18* results in a higher lignin content as measured in the cell walls of the SRDX lines compared to the WT (**Table 1**). Compared to the WT, the expression of genes involved in lignin biosynthesis and polymerization of SRDX lines was higher in young stems, with early developmental steps of secondary growth (upper portion of the stem), but not in the xylem from the stem with well-established secondary growth (lower portion of the stem) (**Figure 6** and **Supplementary Figure 6**). These genes include *PAL2*, the first gene of the monolignol biosynthesis pathway (Wang et al., 2014), *HCT1*, encoding a central enzyme for the direction of the flux towards coniferyl and sinapyl alcohols (Hoffmann et al., 2003), *F5H1* encoding a key enzyme for the synthesis of sinapyl alcohol (Meyer et al., 1998) and a gene homologous to the *A. thaliana peroxidase 72* (*PRX72*), which has been shown to play a role in monolignol polymerization (Herrero et al., 2013). Therefore, the increased lignin accumulation in older xylem tissue of the SRDX lines seems to originate from lignin metabolism activation at earlier stages of secondary growth. Besides, the gene encoding VND6-C1, belonging to the VND6 family of NAC TFs which are master regulators of xylem vessel element differentiation, was upregulated in the SRDX lines (**Supplementary Figure 7**), suggesting that PtaPLATZ18 acts upstream of the complex NAC-MYB network regulating xylogenesis in both Arabidopsis and poplar (Kubo et al., 2005; Ohtani et al., 2011; Li et al., 2012). A more in-depth study through RNA-seq analysis would give a comprehensive overview of the transcriptional changes induced by the alteration of *PtaPLATZ18* expression.

Future research on the transcriptional activity of PtaPLATZ18, *i.e.* as an activator or as a repressor, on its protein interactions, and on the biological processes regulated by this TF are required to unravel its role in plant vegetative growth, secondary growth, and wood formation.

## Data availability statement

The original contributions presented in the study are included in the article/**Supplementary Material**, further inquiries can be directed to the corresponding author. Nucleotide sequence of *PtaPLATZ18* can be found in the GenBank database under the accession number OR730833.

## Author contributions

CG: Conceptualization, Data curation, Formal analysis, Investigation, Methodology, Supervision, Validation, Visualization, Writing – original draft, Writing – review & editing. MBe: Conceptualization, Investigation, Methodology, Supervision, Visualization, Writing – review & editing. JS: Data curation, Formal analysis, Investigation, Validation, Visualization, Writing – original draft. AM: Investigation, Writing – review & editing. ME: Supervision, Writing – review & editing. MBa: Conceptualization, Funding acquisition, Methodology, Project administration, Supervision, Writing – original draft, Writing – review & editing.
