# Supplementary figures and images for: Evidence for poplar PtaPLATZ18 in the regulation of plant growth and vascular tissues development
Source: Front Plant Sci. 2023 Dec 21;14:1302536. doi: 10.3389/fpls.2023.1302536 (PMC10768006; doi:10.3389/fpls.2023.1302536)

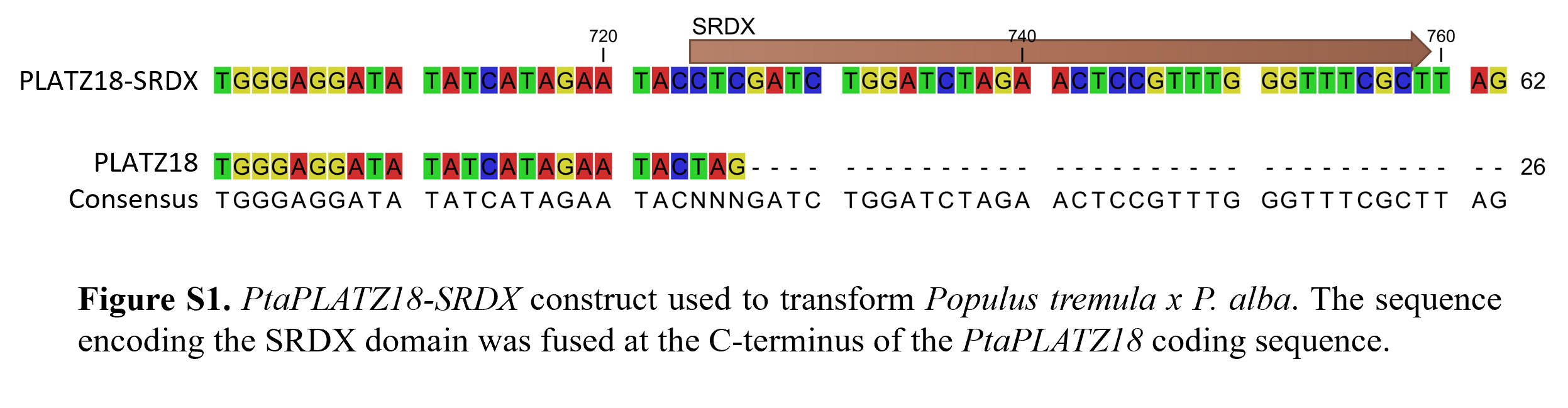

Supplement: Supplementary file 2 [file Image_1.tif]

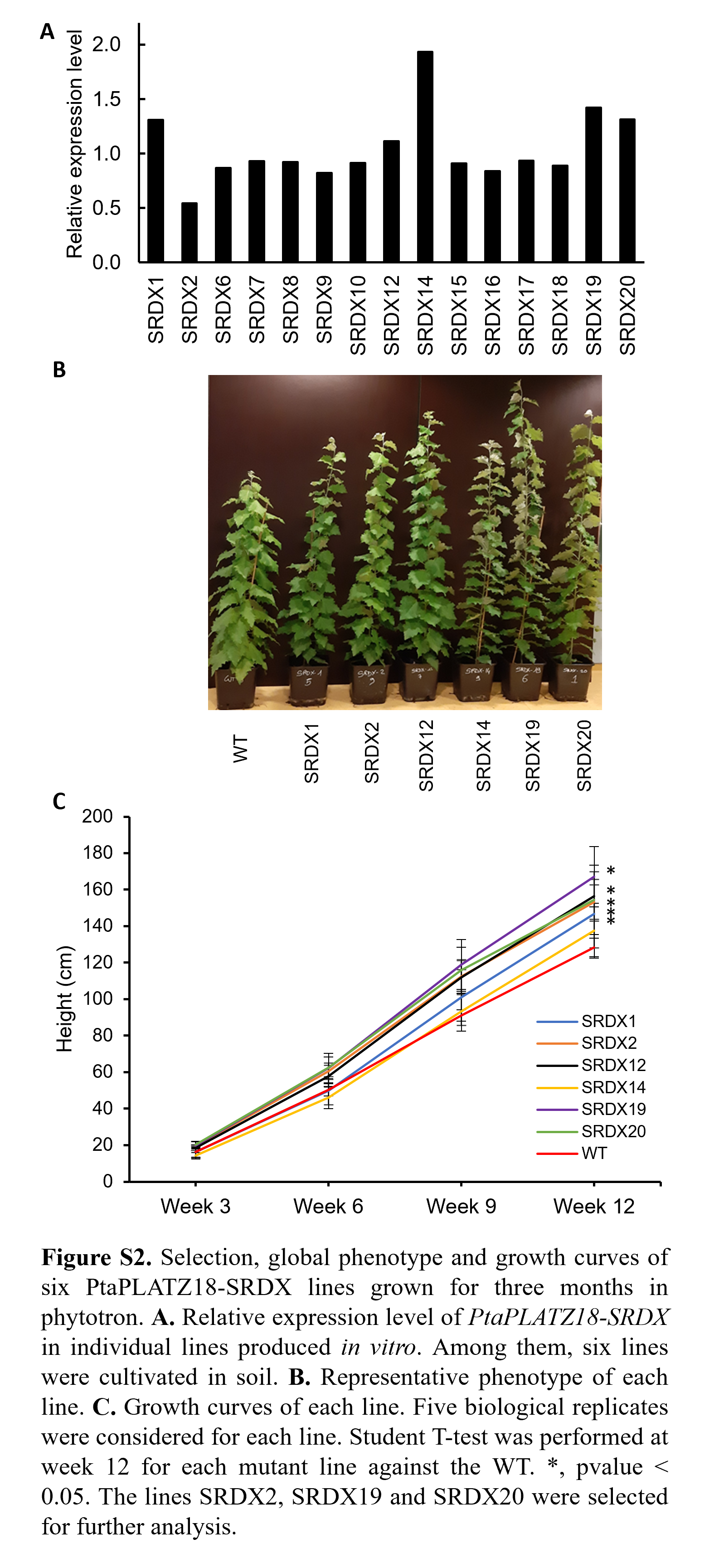

Supplement: Supplementary file 3 [file Image_2.tif]

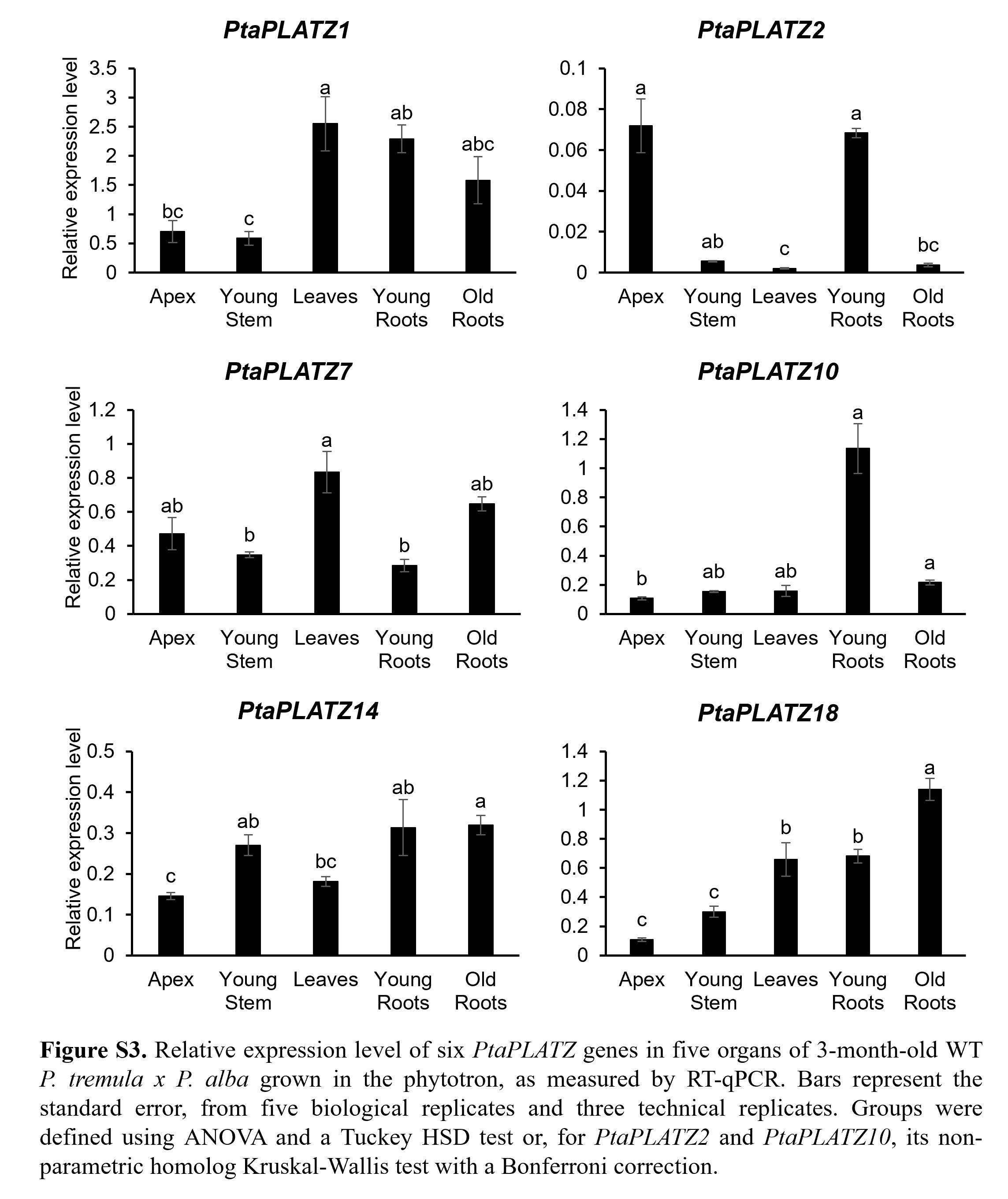

Supplement: Supplementary file 4 [file Image_3.tif]

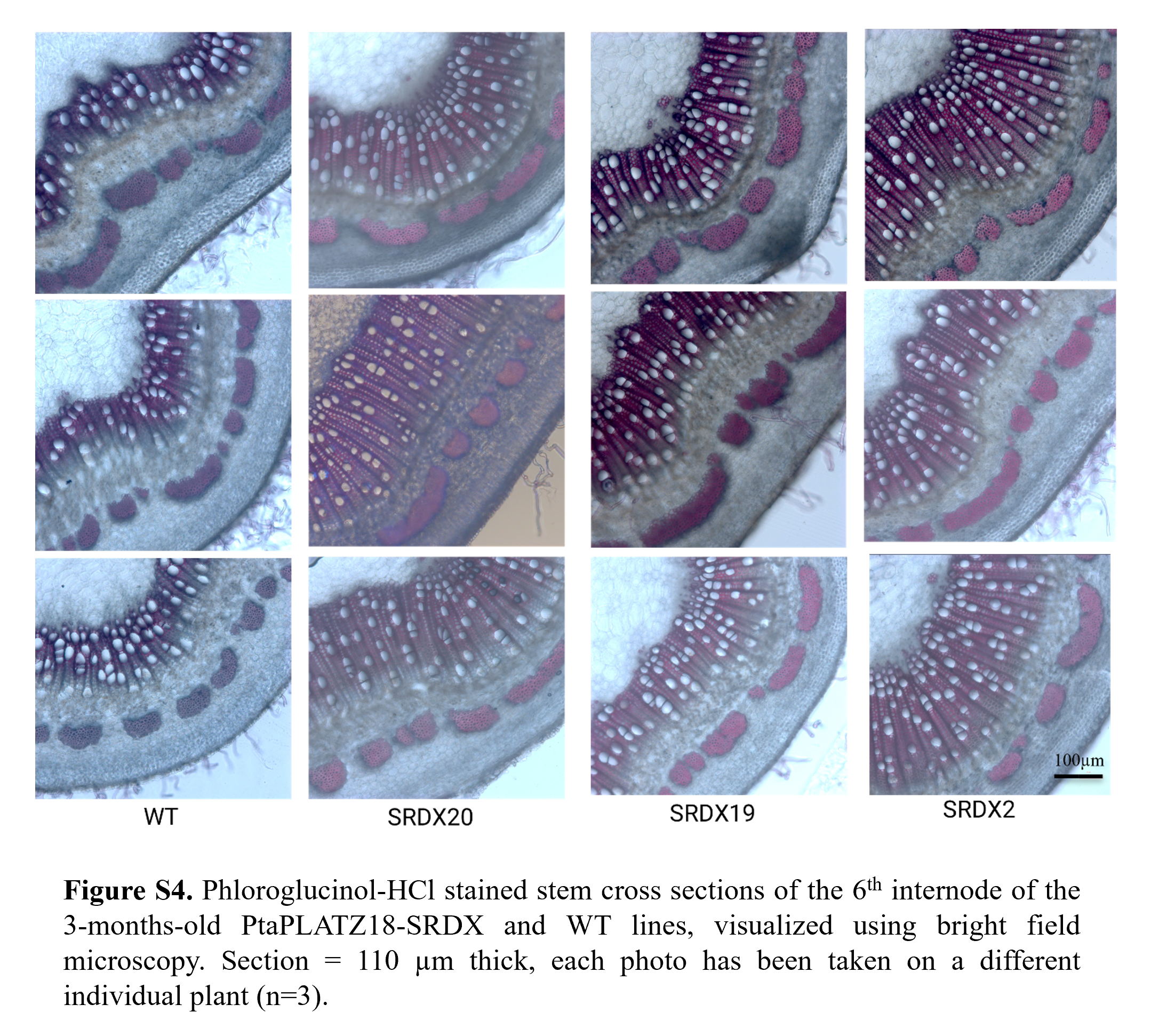

Supplement: Supplementary file 5 [file Image_4.tif]

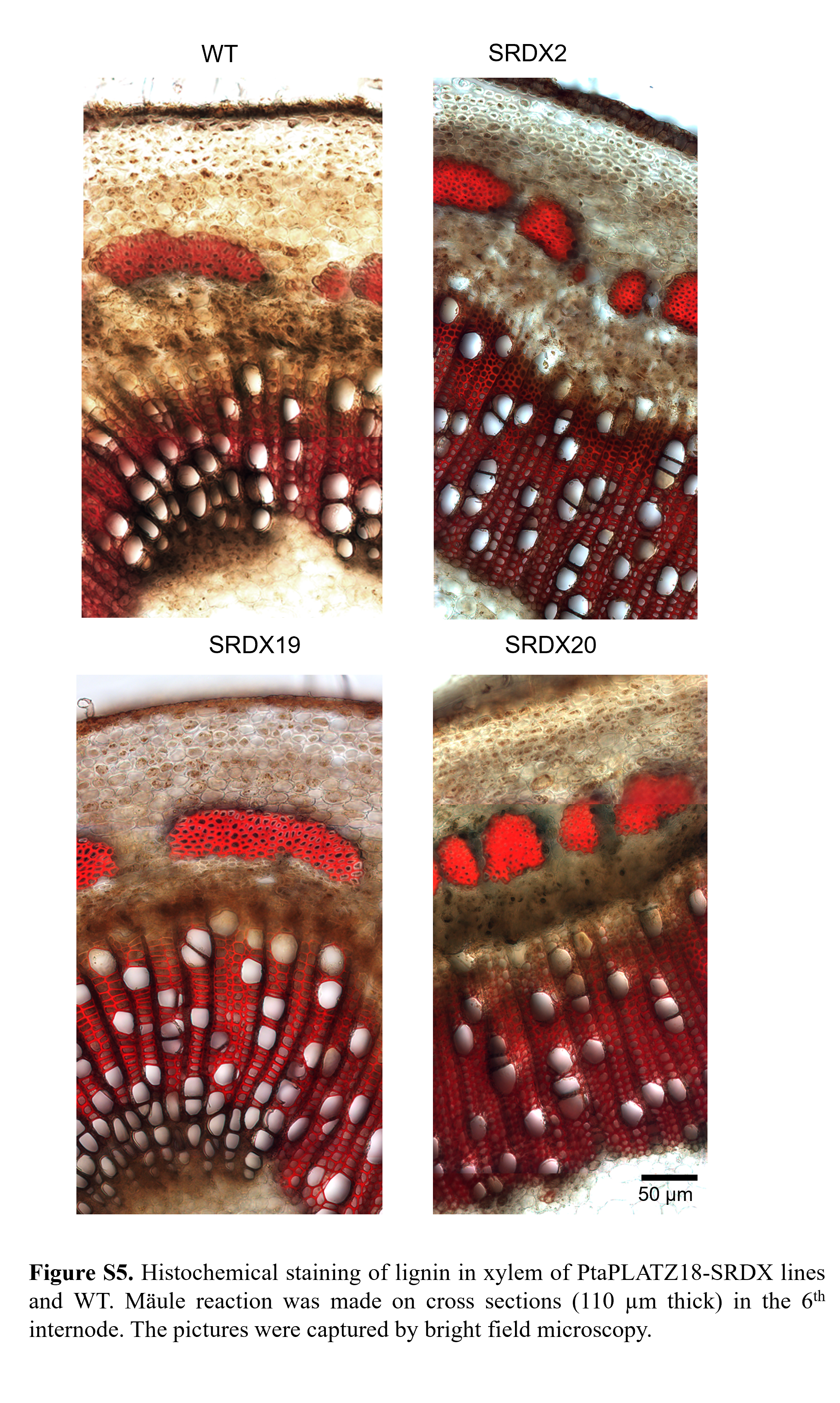

Supplement: Supplementary file 6 [file Image_5.tif]

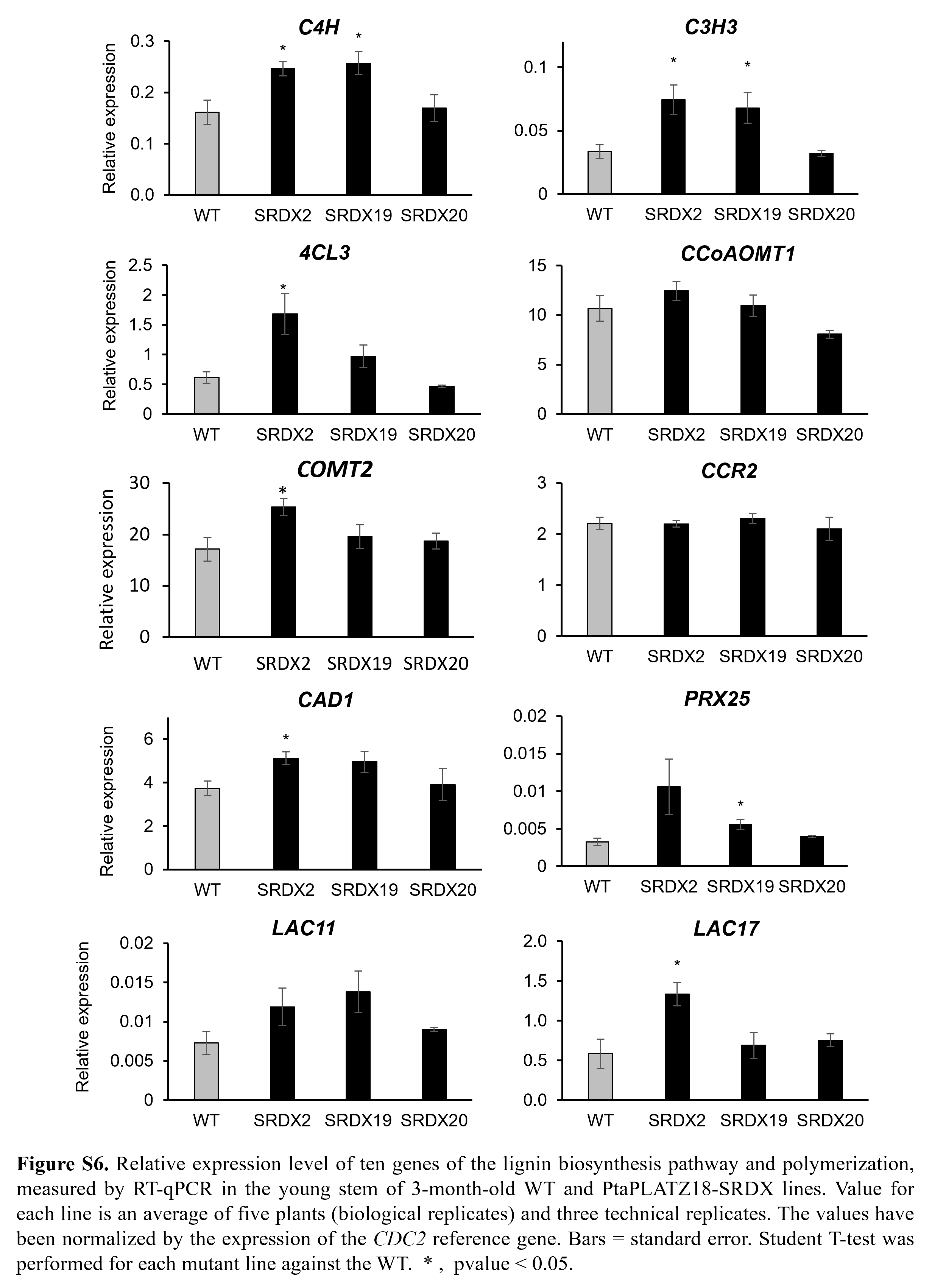

Supplement: Supplementary file 7 [file Image_6.tif]

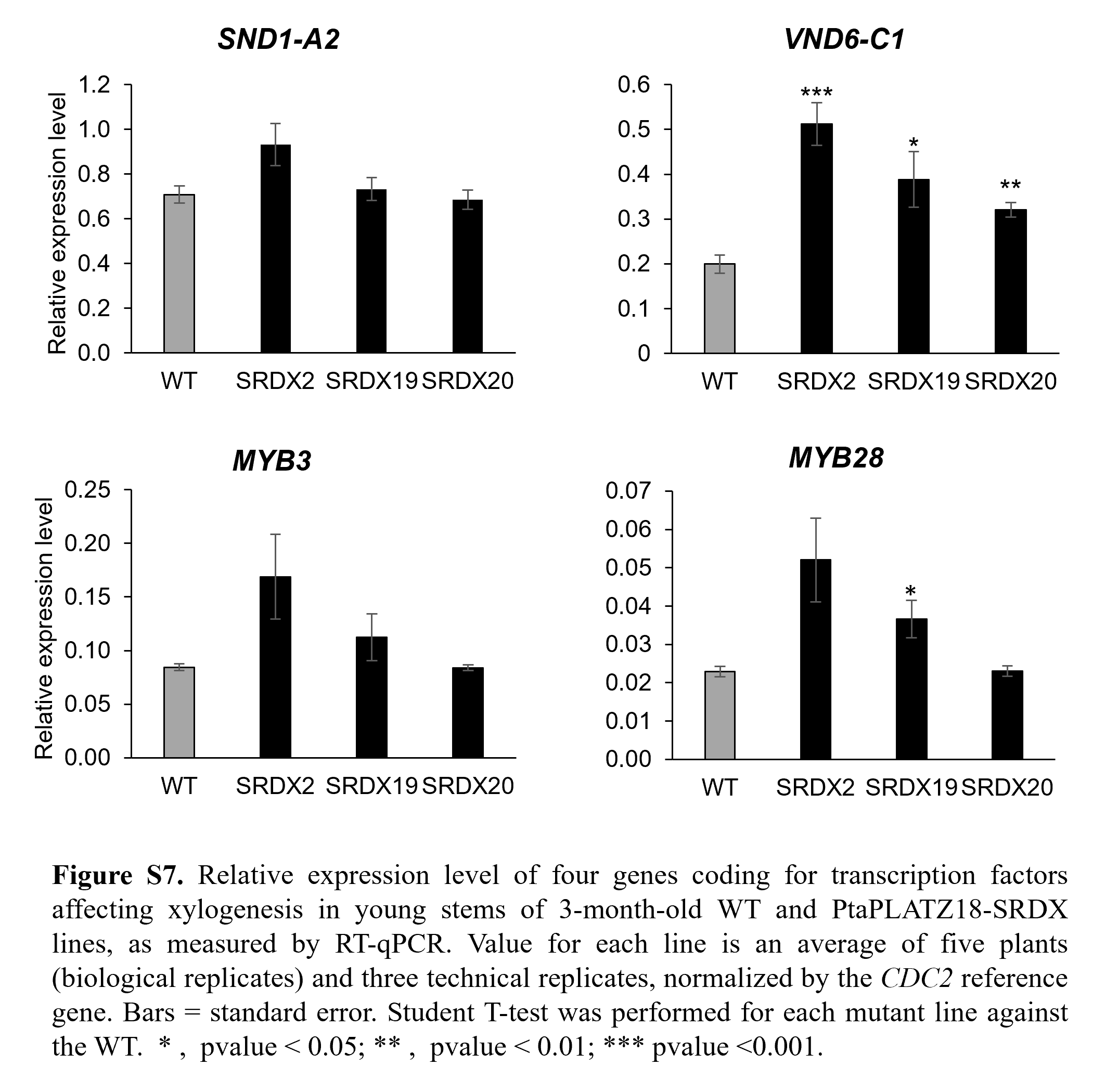

Supplement: Supplementary file 8 [file Image_7.tif]
